# The Comparative Study for Detection of Canine Vector-Borne Pathogens Between Companion and Stray Dogs in Bangkok and Vicinities, Thailand

**DOI:** 10.3390/pathogens15050527

**Published:** 2026-05-14

**Authors:** Bach Xuan Pham, Pornkamol Phoosangwalthong, Techin Inkaew, Tawin Inpankaew

**Affiliations:** 1 Department of Parasitology, Faculty of Veterinary Medicine, Kasetsart University, Bangkok 10900, Thailand; bachxuan.p@ku.th (B.X.P.); pornkamol.ph@ku.th (P.P.); 2 Graduate Program in Animal Health and Biomedical Sciences, Faculty of Veterinary Medicine, Kasetsart University, Bangkok 10900, Thailand; 3 Pinnacle Pet Hospital (Khlong Sam Wa) Bang Chan, Khlong Sam Wa, Bangkok 10510, Thailand; alexbkk2555@gmail.com

**Keywords:** vector-borne pathogen, *Rickettsia*, *Anaplasma*, *Babesia*, *Ehrlichia*, stray dogs, companion dogs, Bangkok

## Abstract

This study investigated the prevalence and molecular characteristics of canine vector-borne pathogens (CVBPs) circulating in diverse dog populations in Thailand by using molecular diagnostic methods. A total of 400 blood samples were collected from four groups (*n* = 100 each): stray dogs (Group A), vector-borne disease–suspected companion dogs (Group B), healthy companion dogs presenting for routine examination (Group C), and companion dogs presenting with non-vector-borne illnesses (Group D). The overall infection rate was 46.25%. *Ehrlichia* spp. were the most frequently detected pathogens (23.5%), followed by *Babesia* spp. (16.5%), *Rickettsia* spp. (15.0%), and *Anaplasma* spp. (11.5%). The prevalence differed markedly among groups, including group A (88.0%), group B (54.0%), group C (27.0%) and group D (16.0%) (*p* < 0.05). DNA sequence analysis showed 100% identity with GenBank™ reference sequences, confirming the presence of *Ehrlichia canis*, *Rickettsia asembonensis*, *Babesia vogeli*, and *Anaplasma platys*. The detection of CVBPs across all groups demonstrates free-roaming and owned dogs serve as reservoirs for substantial ongoing infections and pose potential zoonotic implications to humans. Overall, these findings emphasize the importance of sustained molecular surveillance, improved vector control strategies, and proactive monitoring of high-risk dog populations to reduce the burden of CVBPs in Thailand.

## 1. Introduction

Canine vector-borne diseases (CVBDs) are a significant threat to the health and welfare of both domestic and stray dog populations worldwide [1]. These diseases are caused by a variety of pathogens, including Apicomplexan protozoans (*Babesia* spp. and *Hepatozoon* spp.), Anaplasmataceae (*Anaplasma* spp. and *Ehrlichia* spp.), and other bacterial pathogens such as *Rickettsia* spp., *Mycoplasma* spp., or *Bartonella* spp., which can affect both canines and humans in endemic regions [2]. CVBD agent infections may remain asymptomatic or present non-specific clinical signs until the late stages of disease, challenging early and accurate diagnosis. The clinical signs are characterized by protean manifestations ranging from fever, anorexia, weight loss, lethargy, or even death in severe cases [3]. Evaluating the comparative prevalence of canine vector-borne pathogens (CVBPs) between different dog populations is crucial for understanding the epidemiology of these diseases and implementing effective control strategies. Numerous studies have highlighted the significant impact of stray dogs on the transmission of zoonotic diseases, as they may serve as reservoirs and vectors for a variety of pathogens [4,5]. The surge in the number of dogs and the potential for these animals to acquire and transmit parasitic infections underscores the need for comprehensive surveillance and prevention efforts. Companion dogs, which typically receive sufficient veterinary attention, may have a lower prevalence of these infections compared to semi-domesticated or stray dogs, which often lack access to veterinary care and preventive measures [6].

Thailand has a diverse dog population, including both companion and stray dogs. It provides an ideal location to investigate the comparative prevalence of CVBPs. Studying the epidemic condition, analyzing and comparing the genetic variety of CVBPs would help to manage and avoid outbreaks of these infections in dogs. This study aims to determine the prevalence of *Babesia vogeli*, *Anaplasma platys*, *Erhlichia canis* and *Rickettsia* spp. and analyze the risk factors associated with CVBP infection among different dog groups in Bangkok, Thailand.

## 2. Materials and Methods

### 2.1. Study Area and Sample Collection

A total of 400 blood samples were collected by convenience sampling from June to December 2019. Samples were categorized into four groups (*n* = 100 each): stray dogs (Group A); companion dogs suspected of carrying vector-borne pathogens (Group B); companion dogs presenting for routine health examinations (Group C); and non-vector-related illness dogs (Group D). Stray dog samples were obtained from temples across multiple subdistricts in Bangkok and the surrounding metropolitan area and were used specifically for the purpose of this study. Blood collection from these dogs was performed once per animal by licensed veterinarians or trained veterinary staff, using gentle physical restraint and standard venipuncture techniques. Throughout capture, handling, sampling, and release, measures were taken to minimize stress, pain, and handling time, and animal welfare was prioritized in accordance with institutional, national, and international guidelines for the care and use of animals in research. Client-owned dog samples were collected from three small animal hospitals in Bangkok and vicinities. Group B included dogs exhibiting clinical signs compatible with CVBDs, such as fever, lethargy, anorexia, pale mucous membranes, weight loss, thrombocytopenia and anemia, as determined by the attending veterinarian. Groups C and D consisted of dogs without clinical signs suggestive of CVBDs. Blood samples were collected as part of the animals’ diagnostic work-up or routine health evaluation at the participating veterinary hospitals. Residual EDTA blood that remained after the completion of the requested clinical tests was used for this study, with the written informed consent of the owners, after they were informed about the objectives of the research and the intended use of the samples. All sampling and handling procedures were performed by licensed veterinarians or trained veterinary technicians following standard hospital protocols and national animal welfare guidelines. The protocol for the capture, sampling, and subsequent management of dogs was reviewed and approved by the Kasetsart University’s Institution Animal Care and Use Committee (approval number ACKU60-VET-006), and no invasive procedures beyond those described in the approved protocol were performed.

Data regarding the animals, including sex (male, female), age (<1 year—puppy, 1–5 years—juvenile, >5 year—adult), free-roaming (yes, no), antiparasitic treatment (yes, no), were collected by interviewing the monks, animal caretakers, or pet owners for risk factors analysis. After obtaining the consent, blood samples were collected via cephalic vein into sodium citrate with blood coagulation by veterinarians or veterinary technicians. All samples were stored at −20 °C at the Department of Parasitology, Faculty of Veterinary Medicine, Kasetsart University before being used.

### 2.2. DNA Extraction and Molecular Detection of CVBPs

DNA was extracted from 250 μL of sodium citrate-anticoagulated blood using a commercial DNA extraction kit (E.Z.N.A.^®^ Blood DNA Mini Kit, Omega Biotek Inc., Norcross, GA, USA) according to the manufacturer’ s instructions. The elution volume was reduced to 100 μL and DNA stored at −20 °C until used. Blood DNA samples were tested for the presence of *Babesia vogeli*, *Anaplasma platys*, *Ehrlichia canis*, and *Rickettsia* spp. All primers and PCR protocols used for the detection of CVBPs were summarized in Table 1. For all reactions, pathogen-positive samples were used as positive controls and distilled, deionized water was used as the negative control. DNA of pathogens were identified by electrophoresis with 1.5% agarose gel.

### 2.3. Sequence and Phylogenetic Analysis

Positive PCR products were purified from the gel amplicons using MEGAquick-spin^TM^ plus Fragment DNS purification kit (Intron Biotechnology, Seoul, Republic of Korea). The purified DNA was then sequenced by a commercial company (U2Bio Co., Ltd., Bangkok, Thailand) using Sanger sequencing. Chromatograms were visually inspected and edited in FinchTV (version 1.3.0), low-quality bases (Phrethd score < 20), and both ends of the reads were trimmed. The sequences, therefore, consist only of bases with Phred quality scores ≥20. To identify the pathogen species, the sequences were compared against the U.S. National Center for Biotechnology Information (NCBI) database using the Basic Local Alignment Search Tool (BLAST+ 2.17.0). A phylogenetic tree incorporating the four pathogens was constructed using the Maximum Likelihood method with 1000 bootstrap replications (MEGA 12 software). Each reference sequence in the tree is identified by its GenBank accession number, species name, and country of origin.

### 2.4. Statistical Analysis

Statistical analyses were conducted using SPSS version 27. The prevalence of the infections in dogs was summarized using cross-tabulations. Univariate analysis was performed using the Chi-square test to determine the association between the presence of *Babesia*, *Anaplasma*, *Ehrlichia*, and *Rickettsia* such as sex, age, free-roaming, antiparasitic treatment, and groups. Variables with significant associations in the univariate analysis were further analyzed using a multivariable logistic regression model to calculate adjusted odds ratios (ORs) and 95% confidence intervals (CIs) to evaluate the strength of relationship between each category and infection. Statistical significance was established at *p* ≤ 0.05.

## 3. Results

### 3.1. Prevalence of CVBPs and Co-Infection in Dogs

The overall prevalence of CVBPs in this study was 46.25% (Table 2). *E. canis* was the most frequently identified pathogen (23.5%), followed by *B. vogeli* (16.5%), *R. asembonensis* (15.0%), and *A. platys* (11.5%). Regarding specific cohorts, stray dogs (Group A) had the highest positivity rate at 88.0%, primarily driven by a high frequency of *E. canis* (61.0%) and *R. asembonensis* (49.0%). Companion dogs with a suspected CVBP (Group B) showed a 54.0% positivity rate, with *A. platys* being the most common pathogen (28.0%). Dogs presented for routine examinations (Group C) and those with suspected dog illness (Group D) showed lower prevalence rates of 27.0% and 16.0%, respectively. Statistical analysis indicated that stray dogs and CVBD-suspected dogs had significant higher prevalence than routine examination and non-vector-related illness dogs (*p* < 0.05).

The analysis of co-infection revealed a high degree of complexity (Table 3), particularly among stray dogs (Group A). Co-infection of *R. asembonensis* + *E. canis* was the most prevalent in stray dog group (33.00%), followed by concurrent infections of *B. vogeli* + *E. canis* (15.00%) and *B. vogeli* + *R. asembonensis* (14.00%). Especially, the co-infection of three (17.00%) and four pathogens (1.00%) was also found in stray dogs. All companion dog groups (B, C, and D) exhibited simpler co-infection patterns, limited to two-pathogen combinations, most commonly *B. vogeli* + *E. canis* or *R. asembonensis*, and *E. canis* + *A. platys*.

### 3.2. Genetic Characterization of CVBPs in Dogs

The genetic characterization of CVBPs in dogs were compared to GenBank™ databases. The resulting sequences showed 100% nucleotide identity with reference isolates deposited in GenBank. Specifically, the *Rickettsia* isolates were identified as *Rickettsia asembonensis* (100% identity; KY650699), the *Anaplasma* isolates as *Anaplasma platys* (100% identity; AF478129), the *Babesia* isolates as *Babesia vogeli* (100% identity; MW255605), and the *Ehrlichia* isolates as *Ehrlichia canis* (100% identity; CP025749). Sequence length, coverage, and quality metrics for each pathogen are presented in Table 4.

**Figure 1:**
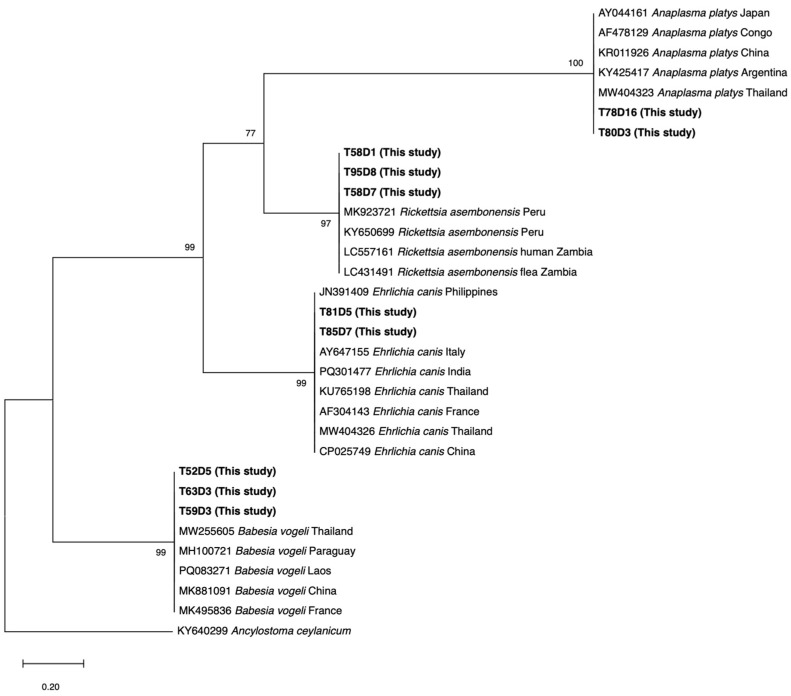
Phylogenetic analysis of four pathogen sequences based on groEL gene (*A. platys*), gltA gene (*E. canis*), ompB (*R. asembonensis*), and 18s rRNA (*B. vogeli*) obtained from this research using Neighbor-Joining Tree (Kimura-two model). Isolates obtained from this study are presented in bold text.

Phylogenetic analysis of the obtained sequences revealed four distinct, well-supported clades, representing major CVBP circulation in this study (Figure 1). Study samples T78D16 and T80D3 clustered with 100% bootstrap support within the *Anaplasma platys* lineage, showing high identity to reference strains from Thailand and various global regions. Samples T58D1, T95D8, and T58D7 formed a monophyletic group with *Rickettsia* sequences (bootstrap 97%), closely related to the *R. asembonensis*. The detection of *Ehrlichia canis* was confirmed by the clustering of samples T81D5 and T85D7 with international and local isolates at a 99% bootstrap level, highlighting the genetic conservation of this pathogen. Furthermore, samples T52D5, T63D3, and T59D3 were identified as *Babesia vogeli*, grouping strongly (99%) with reported references.

### 3.3. Risk Factors Associated with CVBP Infections in Dogs

The analysis of risk factors was presented in Table 5. In the univariate analysis, sex was not associated with CVBP infection, with similar proportions of positives in males (45.5%) and females (47.4%; *p* = 0.604). Age showed a significant overall association (*p* = 0.028), with higher infection percentages in dogs <1 year (54.4%) and 1–5 years (49.4%) than in dogs >5 years (38.0%). Roaming status was strongly associated with infection in univariate analysis, with 52.8% positivity in free-roaming dogs versus 29.5% in non–free-roaming dogs (*p* < 0.001). Dogs without antiparasitic treatment also had a higher prevalence (62.2% vs. 30.9%; *p* < 0.001). Infection proportions differed markedly by group (*p* < 0.001), being the highest in stray dogs (group A, 88.0%), followed by CVBD-suspected dogs (group B, 54.0%), routine examination dogs (group C, 27.0%), and non-vector-borne-related dogs (group D, 16.0%). Multivariate logistic regression revealed none of the sex, age categories, free-roaming status, or antiparasitic treatment showed a statistically significant adjusted odds ratio (*p* > 0.05). By contrast, dog group remained significant after adjustment. The risk of CVBP infection was significantly higher in Group A, with the odds being 28.88 times greater than those of Group D (95% CI: 12.35–67.54; *p* < 0.001). Similarly, Group B exhibited 5.78 times the odds of infection compared to Group D (95% CI: 2.95–11.34; *p* < 0.001).

## 4. Discussion

The study revealed a high overall prevalence of CVBPs of 46.25%, indicating the endemic condition of detected pathogens in Bangkok’s dog populations. Our detection rate of CVBPs is consistent with previously reported prevalence in different dog populations in the region, with approximately 40–78% of positive animals [10,11,12]. Thailand weather is characterized by hot and humid condition, providing suitable condition for vector proliferation [13]. Especially, the brown dog ticks (*Rhipicephalus sanguineus*) are the most dominant ectoparasite that primarily target dogs and they serve as the intermediate vector for numerous CVBDs in Southeast Asia [14,15,16].

This current research observed significantly higher infection rate in stray dogs and CVBD-suspected companion dogs compared to the other two groups. Regarding the stray dogs involved in our study, they typically roamed freely around their living area and received little or no ectoparasite control, which substantially increases the likelihood of exposure to multiple vector species and, consequently, to CVBPs. Published studies in endemic areas consistently reported higher CVBP prevalence in unmanaged, outdoor dogs compared to owned pets with regular prophylaxis [17,18], which support elevated OR (28.8) found in stray dogs of this study. Dogs clinically suspected of CVBDs also showed a high adjusted ORs (5.78), which was expected given their presentation of hallmark symptoms such as fever, lethargy, pale mucous membrane and hematological abnormalities, resulting in a strong association between their clinical status and molecular confirmation of CVBPs. Since CVBPs were detected across all groups, it indicated that clinically healthy dogs can also serve as infection reservoirs to spread the diseases to other individuals [19]. In clinical settings, the diagnosis of vector-borne diseases usually relies on conventional methods, including blood smears, complete blood count, or clinical signs; therefore, misdiagnosis is inevitable [10]. Therefore, molecular-based diagnosis is essential for diagnosing the current level of CVBP infection and it is necessary to screen multiple pathogens for higher diagnostic accuracy.

*E. canis* was the most dominant pathogen in surveyed dogs, accounting for 23.5%. This pathogen has been found with high prevalence in several Southeast Asia countries such as Vietnam, Cambodia and the Philippines [12,16,20]. This current study is relevant to previous Bangkok temple-dog survey where *E. canis* was one of the most frequently detected tick-borne pathogens [21]. In Thailand, the infection of *A. platys* is considered as endemic as the previously reported molecular-based prevalence was around 5–30%, depending on different regions and diagnostic methods [22,23,24]. Therefore, the infection rate of *A. platys* (11.5%) in this study is relevant with the previous reports in the country. Moreover, 16.5% of *B. vogeli* infection indicated that canine babesiosis represents a substantial component of the local vector-borne disease burden. This level of infection is consistent with the previous molecular study in Bangkok temple dogs (18.1% positive) [21]. Taken together, the concurrent detection of *E. canis*, *A. platys*, and *B. vogeli* suggests sustained exposure of the study population to the brown dog tick, *Rhipicephalus sanguineus sensu lato*, which is recognized as the principal vector of these pathogens in dogs in Thailand [25]. Dogs serve as reservoir for *Rickettsia* spp., potentially facilitating transmission to humans through arthropod bites [15]. Canine rickettsiosis has occurred in several SEA countries, including Indonesia, Laos, Malaysia, the Philippines, Thailand, and Vietnam [16]. Especially, zoonotic transmission has also been documented, with human infections reported in Thailand, Laos, Vietnam, Indonesia [26,27,28]. While canine rickettsiosis was previously associated with *R. felis*, recent research has revealed that *R. asembonensis* is the most prevalent rickettsial species found in dogs and their fleas [29]. Due to close genetic similarity, *R. asembonensis* is considered as *R. felis*-like organism; however, its role as a primary causative agent of clinical disease in dogs is still under investigation [30]. Taken together, the substantial prevalence of CVBPs across both stray and companion dog populations underscores the necessity of sustained molecular surveillance, regular tick and flea control, and targeted monitoring of high-risk groups to mitigate the overall burden of CVBDs in Thailand.

Previously in Thailand, cases of co-infection with up to four vector-borne pathogens were reported, highlighting the complexity of canine tick-borne disease transmission in endemic settings [21]. In the present study, co-infection patterns were also substantial and varied by dog group, with the greatest complexity observed in stray dogs. By contrast, companion dog groups B, C, and D showed simpler co-infection profiles that were limited to two-pathogen combinations, which is broadly consistent with a recent report from central Thailand describing mixed infections of *Babesia*, *Ehrlichia*, and *Hepatozoon* in owned dogs at a relatively low overall frequency [31]. Co-infection in dogs may arise either from a single vector carrying more than one pathogen or from repeated exposure to multiple infected vectors over time, particularly in tropical environments where infestation with *R. sanguineus* is common and persistent [10]. Despite the high incidence of co-infection, especially in stray dogs, the majority of dogs with concurrent infections showed mild or no clinical signs to hemoparasite infections. In endemic settings, dogs could repeatedly be exposed to local vector-borne pathogens and often develop partial immunity, leading to subclinical or milder infections in many chronically exposed animals [11].

Several limitations could be pointed out from this study. Firstly, the cross-sectional design and convenience sampling of dogs, which was primarily from temples and a select number of veterinary hospitals, may limit the generalizability of the findings to the broader dog population in Bangkok. Secondly, while PCR- and single-direction Sanger sequencing were sufficient for species identification, this approach may underestimate within-species genetic diversity and the full complexity of co-infections, especially for *Rickettsia* spp. identification. Finally, the lack of systematic clinical and hematological analysis restricted our ability to correlate specific pathogens with disease severity or clinical outcomes.

## 5. Conclusions

The overall prevalence of canine vector-borne pathogen infections in the study population was 46.25%, with *Ehrlichia* identified as the most prevalent species at 23.5%. Genetic characterization further revealed that the pathogens circulating among the surveyed dogs included *Ehrlichia canis*, *Rickettsia asembonensis*, *Babesia vogeli*, and *Anaplasma platys*. In addition, this research found that stray dogs and dogs presented with compatible clinical signs to CVBDs have a significant risk of CVBP infections. There should be additional studies that emphasize the importance of proactive surveys in order to better understand the complexity of the distribution of CVBPs in dogs as carriers of disease in Thailand to lead to planning, control and prevention of CVBP infection in dogs.

## Figures and Tables

**Table 1:** Primers used for pathogen detection in this study.

Pathogen	Primer (5′–3′)	Product Size (bp)	Target Gene	PCR Protocol			References
*Rickettsia* spp.	CGACGTTAACGGTTTCTCATTCT ACCGGTTTCTTTGTAGTTTTCGTC	297	*ompB*	95 °C	15 m		[7]
				95 °C	15 s	40×	
				54 °C	30 s		
				72 °C	30 s		
				72 °C	7 m		
*Anaplasma platys*	AAGGCGAAAGAAGCAGTCTTA CATAGTCTGAAGTGGAGGAC	724	*groEL*	95 °C	15 m		[8]
				94 °C	15 s	35×	
				50 °C	30 s		
				72 °C	30 s		
				72 °C	2 m		
*Ehrlichia canis*	TTATCTGTTTATGTTATATAAGC CAGTACCTATGCATATCAATCC	1372	*gltA*	95 °C	15 m		[9]
				95 °C	15 s	35×	
				51 °C	30 s		
				72 °C	30s		
				72 °C	2 m		
*Babesia vogeli*	GTTTATTAGTTTGAAACCCGC GAACTCGAAAAAGCCAAACGA	455	*18S rRNA*	95 °C	15 m		[9]
				95 °C	30 s	35×	
				60 °C	30 s		
				72 °C	30 s		
				72 °C	5 m		

*ompB*: outer membrane protein B, *groEL*: heat shock protein gene, *gltA*: citrate synthase gene.

**Table 2:** The overall prevalence of CVBPs in different groups.

Pathogens	Infected Dogs (%)				Total (%)
	Group A (Stray Dogs)	Group B (CVBD-Suspected Dogs)	Group C (Routine Examination Dogs)	Group D (Non-Vector-Related Illness Dogs)	
*Babesia vogeli*	28/100 (28.00)	16/100 (16.00)	14/100 (14.00)	8/100 (8.00)	66/400 (16.50)
*Anaplasma platys*	12/100 (12.00)	28/100 (28.00)	5/100 (5.00)	1/100 (1.00)	46/400 (11.50)
*Ehrlichia canis*	61/100 (61.00)	21/100 (21.00)	4/100 (4.00)	8/100 (8.00)	94/400 (23.50)
*Rickettsia asembonensis*	49/100 (49.00)	2/100 (2.00)	7/100 (7.00)	2/100 (2.00)	60/400 (15.00)
Total	88/100 ^a^ (88.00)	54/100 ^b^ (54.00)	27/100 ^c^ (27.00)	16/100 ^c^ (16.00)	185/400 (46.25)

Values within the same row with different superscript letters (^a^, ^b^, ^c^) are significantly different (*p* < 0.05) based on Chi-square post hoc comparison with Bonferroni correction.

**Table 3:** The occurrence of CVBPs in dogs with co-infection.

Group	Pathogens Detected	Positive (%)
A (Stray dogs)	Ba + Ri	14 (14.00)
	Ri + Eh	33 (33.0)
	Ri + An	8 (8.00)
	Ba + An	2 (2.00)
	Ba + Eh	15 (15.00)
	Eh + An	6 (6.00)
	Ri + Ba + Eh	9 (9.00)
	Ri + Ba + An	2 (2.00)
	Ba + Eh + An	1 (1.00)
	Eh + An + Ri	5 (5.00)
	Ri + Ba + Eh + An	1 (1.00)
B (CVBD-suspected dogs)	Ba + Ri	1 (1.00)
	Ri + Eh	1 (1.00)
	Ba + An	1 (1.00)
C (Routine examination dogs)	Ba + Ri	1 (1.00)
	Ba + Eh	2 (2.00)
D (Non-vector-related illness dogs)	Ba + Ri	2 (2.00)
	Ba + An	1 (1.00)
	Ba + Eh	3 (3.00)
	Eh + An	7 (7.00)

Ba = *B. vogeli*, An = *A. platys*, Eh = *E. canis*, Ri = *R. asembonensis*.

**Table 4:** Genetic characterization of CVBPs in dogs.

Pathogen Detected	Compared Species on GenBank	Accession Number	Level of Similarity	Sequence Length	Coverage	Quality Metrics
*Babesia* spp.	*B. vogeli*	MW255605	100%	353 bp	Partial	Q ≥ 20
*Anaplasma* spp.	*A. platys*	AF478129	100%	650 bp	Partial	Q ≥ 20
*Ehrlichia* spp.	*E. canis*	CP025749	100%	987 bp	Partial	Q ≥ 20
*Rickettsia* spp.	*R. asembonensis*	KY650699	100%	237 bp	Partial	Q ≥ 20

**Table 5:** Risk factors associated with CVBP infections.

Factor	No. Dogs	No. Positive (%)	χ^2^	*p*-Value	Adjusted OR (95% CI)	*p*-Value
**Sex**			1.009	0.604		
Male	209	95 (45.5)				
Female	191	90 (47.4)				
**Age**			7.166	0.028		
<1 year	90	49 (54.4)			1.36 (0.72–2.57)	0.336
1–5 year	160	79 (49.4)			1.06 (0.61–1.86)	0.837
>5 year	150	57 (38.0)			Ref.	
**Free-roaming**			17.631	<0.001		
Yes	288	152 (52.8)			1.32 (0.76–2.29)	0.327
No	112	33 (29.5)			Ref.	
**Antiparasitic treatment**			39.551	<0.001		
No	196	122 (62.2)			1.468 (0.85–2.52)	0.165
Yes	204	63 (30.9)			Ref.	
**Group**			124.249	<0.001		
Group A	100	88 (88.00)			28.88 (12.35–67.54)	<0.001
Group B	100	54 (54.00)			5.78 (2.95–11.34)	<0.001
Group C	100	27 (27.00)			0.92 (0.49–1.71)	0.04
Group D	100	16 (16.00)			Ref.	

Ref. = reference.

## Data Availability

The original contributions presented in this study are included in the article. Further inquiries can be directed to the corresponding author.

## Abbreviations

The following abbreviations are used in this manuscript:

CVBDs	Canine vector-borne diseases
CVBPs	Canine vector-borne pathogens
PCR	Polymerase Chain Reaction

## References

[1] Colwell D.D., Dantas-Torres F., Otranto D. (2011). Vector-borne parasitic zoonoses: Emerging scenarios and new perspectives. Vet. Parasitol..

[2] Kaewmongkol G., Rukkwamsuk T., Sirinarumitr T., Songserm T., Tipsawake S., Jittapalapong S. (2004). A prevalence of *Babesia canis* in stray dogs in Bangkok using PCR technique. J. Kasetsart Vet..

[3] Gülanber A., Gorenflot A., Schetters T.P.M., Carcy B. (2006). First molecular diagnosis of *Babesia vogeli* in domestic dogs from Turkey. Vet. Parasitol..

[4] Chomel B.B. (2014). Emerging and Re-Emerging Zoonoses of Dogs and Cats. Animals.

[5] Guven E., Avcioglu H., Cengiz S., Hayirli A. (2017). Vector-Borne Pathogens in Stray Dogs in Northeastern Turkey. Vector-Borne Zoonotic Dis..

[6] Cortez-Aguirre G.R., Jiménez-Coello M., Gutiérrez-Blanco E., Ortega-Pacheco A. (2018). Stray Dog Population in a City of Southern Mexico and Its Impact on the Contamination of Public Areas. Vet. Med. Int..

[7] Hii S.F., Lawrence A.L., Cuttell L., Tynas R., Abd Rani P.A.M., Šlapeta J., Traub R.J. (2015). Evidence for a specific host-endosymbiont relationship between “*Rickettsia* sp. genotype RF2125” and *Ctenocephalides felis orientis* infesting dogs in India. Parasites Vectors.

[8] Inokuma H., Fujii K., Matsumoto K., Okuda M., Nakagome K., Kosugi R., Hirakawa M., Onishi T. (2002). Demonstration of *Anaplasma* (*Ehrlichia*) platys inclusions in peripheral blood platelets of a dog in Japan. Vet. Parasitol..

[9] Inokuma H., Brouqui P., Drancourt M., Raoult D. (2001). Citrate synthase gene sequence: A new tool for phylogenetic analysis and identification of *Ehrlichia*. J. Clin. Microbiol..

[10] Junsiri W., Taweethavonsawat P. (2025). Comparative analysis of diagnostic methods and genetic analysis for canine vector-borne diseases in Thailand. Sci. Rep..

[11] Dordio A.M., Beck R., Nunes T., Pereira da Fonseca I., Gomes J. (2021). Molecular survey of vector-borne diseases in two groups of domestic dogs from Lisbon, Portugal. Parasites Vectors.

[12] Inpankaew T., Hii S.F., Chimnoi W., Traub R.J. (2016). Canine vector-borne pathogens in semi-domesticated dogs residing in northern Cambodia. Parasites Vectors.

[13] Sweatman G.K. (1967). Physical and biological factors affecting the longevity and oviposition of engorged *Rhipicephalus sanguineus* female ticks. J. Parasitol..

[14] Colella V., Nguyen V.L., Tan D.Y., Lu N., Fang F., Zhijuan Y., Wang J., Liu X., Chen X., Dong J. (2020). Zoonotic vectorborne pathogens and ectoparasites of dogs and cats in Eastern and Southeast Asia. Emerg. Infect. Dis..

[15] Eamudomkarn C., Pitaksakulrat O., Boueroy P., Thanasuwan S., Watwiengkam N., Artchayasawat A., Boonmars T. (2022). Prevalence of *Ehrlichia*-, *Babesia*-, and *Hepatozoon*-infected brown dog ticks in Khon Kaen Province, Northeast Thailand. Vet. World.

[16] Do T., Bui L.K., Umemiya-Shirafuji R., Inpankaew T., Hasan T., Zafar I., Ma Z., Hang L., Mohanta U.K., Amer M. (2024). The detection of zoonotic microorganisms in *Rhipicephalus sanguineus* (brown dog ticks) from Vietnam and the frequency of tick infestations in owned dogs. Front. Vet. Sci..

[17] Cevidanes A., Di Cataldo S., Muñoz-San Martín C., Latrofa M.S., Hernández C., Cattan P.E., Otranto D., Millán J. (2023). Co-infection patterns of vector-borne zoonotic pathogens in owned free-ranging dogs in central Chile. Vet. Res. Commun..

[18] Angelou A., Gelasakis A.I., Verde N., Pantchev N., Schaper R., Chandrashekar R., Papadopoulos E. (2019). Prevalence and risk factors for selected canine vector-borne diseases in Greece. Parasites Vectors.

[19] Manathunga T., Carbonara M., Nekouei O., Mendoza-Roldan J.A., Tam W.Y.J., Beugnet F., Otranto D., Barrs V.R. (2025). High prevalence of vector-borne pathogens in the blood of clinically healthy dogs in Hong Kong. Parasites Vectors.

[20] Galay R.L., Manalo A.A.L., Dolores S.L.D., Aguilar I.P.M., Sandalo K.A.C., Cruz K.B., Divina B.P., Andoh M., Masatani T., Tanaka T. (2018). Molecular detection of tick-borne pathogens in canine population and *Rhipicephalus sanguineus* (sensu lato) ticks from southern Metro Manila and Laguna, Philippines. Parasites Vectors.

[21] Do T., Phoosangwalthong P., Kamyingkird K., Kengradomkij C., Chimnoi W., Inpankaew T. (2021). Molecular Detection of Tick-Borne Pathogens in Stray Dogs and *Rhipicephalus sanguineus* sensu lato Ticks from Bangkok, Thailand. Pathogens.

[22] Piratae S., Senawong P., Chalermchat P., Harnarsa W., Sae-chue B. (2019). Molecular evidence of *Ehrlichia canis* and *Anaplasma platys* and the association of infections with hematological responses in naturally infected dogs in Kalasin, Thailand. Vet. World.

[23] Piratae S., Khiewkham N., Maungmungkun N., Tippornwong C., Seerintra T., Thanasuwan S., Thi Phung L. (2023). Investigation of *Ehrlichia canis* and *Anaplasma platys* from Dogs in Thailand, with Molecular Characterization and Haematological Profiles. Adv. Anim. Vet. Sci..

[24] Purisarn A., Wichianchot S., Maneeruttanarungroj C., Mangkit B., Raksajit W., Kaewmongkol S., Jarudecha T., Sricharern W., Rucksaken R. (2022). Molecular detection and phylogeny of *Ehrlichia canis* and *Anaplasma platys* in naturally infected dogs in Central and Northeast Thailand. Vet. World.

[25] Chimnoi W., Pinyopanuwat N., Kengradomkij C., Inpankaew T., Sinking P., Saengow S., Yangtara S., Suraruangchai D., Sathaporn Jittapalapong S. (2017). Prevalence of external parasites of stray cats and dogs residing in monasteries of Bangkok Metropolitan Areas, Thailand. Proceedings of the 55th Kasetsart University Annual Conference, Bangkok, Thailand.

[26] Edouard S., Bhengsri S., Dowell S.F., Watt G., Parola P., Raoult D. (2014). Two Human Cases of *Rickettsia felis* Infection, Thailand. Emerg. Infect. Dis..

[27] Dittrich S., Phommasone K., Anantatat T., Panyanivong P., Slesak G., Blacksell S.D., Dubot-Pérès A., Castonguay-Vanier J., Stenos J., Newton P.N. (2014). *Rickettsia felis* Infections and Comorbid Conditions, Laos, 2003–2011. Emerg. Infect. Dis..

[28] Le-Viet N., Le V.N., Chung H., Phan D.T., Phan Q.D., Cao T.V., Abat C., Raoult D., Parola P. (2019). Prospective case-control analysis of the aetiologies of acute undifferentiated fever in Vietnam. Emerg. Microbes Infect..

[29] Jiang J., Maina A.N., Knobel D.L., Cleaveland S., Laudisoit A., Wamburu K., Ogola E., Parola P., Breiman R.F., Njenga M.K. (2013). Molecular detection of *Rickettsia felis* and *Candidatus Rickettsia asemboensis* in Fleas from Human Habitats, Asembo, Kenya. Vector-Borne Zoonotic Dis..

[30] Maina A.N., Jiang J., Luce-Fedrow A., John H.K.S., Farris C.M., Richards A.L. (2019). Worldwide Presence and Features of Flea-Borne *Rickettsia asembonensis*. Front. Vet. Sci..

[31] Chamsai T., Saechin A., Mongkolphan C., Sariya L., Tangsudjai S. (2024). Tick-borne pathogens *Ehrlichia*, *Hepatozoon*, and *Babesia* co-infection in owned dogs in Central Thailand. Front. Vet. Sci..

